# THE IMPACT OF FUNCTIONAL DISABILITY ON HOSPITALIZATION SPENDING IN SINGAPORE

**DOI:** 10.1093/geroni/igz038.3083

**Published:** 2019-11-08

**Authors:** Cynthia Chen

**Affiliations:** Saw Swee Hock School of Public Health, Singapore, Singapore, Singapore

## Abstract

Singapore is one of the fastest-aging populations due to increased life expectancy and lowered fertility. Lifestyle changes increase the burden of chronic diseases and disability. These have important implications for social protection systems. The goal of this paper is to model future functional disability and healthcare expenditures based on current trends. To project the health, disability and hospitalization spending of future elders, we adapted the Future Elderly Model (FEM) to Singapore. The FEM is a dynamic Markov microsimulation model developed in the US. Our main source of population data was the Singapore Chinese Health Study (SCHS) consisting of 63,000 respondents followed up over three waves from 1993 to 2010. The FEM model enables us to investigate the effects of disability compounded over the lifecycle and hospitalization spending, while adjusting for competing risk of multi-comorbidities. Results indicate that by 2050, 1 in 6 older adults will have at least one ADL disability and 1 in 3 older adults will have at least one IADL disability, an increase from 1 in 12 elders and 1 in 5 elders respectively in 2014. The highest prevalence of functional disability will be in those aged 85 years and above. Lifetime hospitalization spending of elders aged 55 and above is US$24,400 (30.2%) higher among people with functional disability compared to those without disability. Policies that successfully tackle diabetes and promote healthy living may reduce or delay the onset of disability, leading to potential saving. In addition, further technological improvements may reduce the financial burden of disability.

